# Biopolymer-Based Microencapsulation of Procyanidins from Litchi Peel and Coffee Pulp: Characterization, Bioactivity Preservation, and Stability During Simulated Gastrointestinal Digestion

**DOI:** 10.3390/polym17050687

**Published:** 2025-03-04

**Authors:** María de los Ángeles Vázquez-Nuñez, Nuria E. Rocha-Guzmán, Pedro Aguilar-Zárate, Romeo Rojas, Guillermo Cristian G. Martínez-Ávila, Abigail Reyes, Mariela R. Michel

**Affiliations:** 1 Facultad de Estudios Profesionales Zona Huasteca, Universidad Autónoma de San Luis Potosí, Romualdo del Campo 501, Colonia Rafael Curiel, Ciudad Valles 79060, San Luis Potosí, Mexico; angeles.vazquezac@outlook.com (M.d.l.Á.V.-N.); abigail.reyes@uaslp.mx (A.R.); 2 Laboratorio Nacional CONAHCYT de Apoyo a la Evaluación de Productos Bióticos, Unidad de Servicio Tecnológico Nacional de México/I.T. de Durango, Felipe Pescador 1830 Ote., Durango 34080, Durango, Mexico; nrocha@itdurango.edu.mx; 3 Laboratorio Nacional CONAHCYT de Apoyo a la Evaluación de Productos Bióticos, Unidad de Servicio Tecnológico Nacional de México/I. T. de Ciudad Valles, Carretera al Ingenio Plan de Ayala km 2, Colonia Vista Hermosa, Ciudad Valles 79010, San Luis Potosí, Mexico; 4 Laboratory of Chemistry and Biochemistry, School of Agronomy, Autonomous University of Nuevo Leon, Francisco Villa S/N, Ex Hacienda El Canadá, General Escobedo 66050, Nuevo Leon, Mexico; romeo.rojasmln@uanl.edu.mx (R.R.); guillermo.martinezavl@uanl.edu.mx (G.C.G.M.-Á.)

**Keywords:** flash chromatography, spray drying, condensed tannins, UPLC, microencapsulation, procyanidins digestion, in vitro digestion, in vitro absorption

## Abstract

The need for encapsulation processes in compounds such as procyanidins (PCs) is related to their functional stability, which may limit their application in functional foods. The aim of this study was to evaluate the in vitro digestion of microencapsulated PCs from litchi peel and coffee pulp to determine concentration changes and antioxidant activity. The PCs were extracted, purified, encapsulated, and subjected to in vitro digestion and absorption. Phenolic acids, flavonoids, and PCs were characterized by UPLC-PDA-ESI-QqQ, identifying 27 compounds, including PCs with mean degrees of polymerization (mDP) of 1.2 and 1.7 for lychee and coffee. It was shown that the concentrations of encapsulated PCs were adequately retained during digestion (94.81 ± 4.83 and 90.74 ± 1.77%, lychee and coffee, respectively), with variation in their antioxidant capacity (68.33 ± 2.89 and 77.07 ± 4.59%); however, they showed better results than in their free form. Additionally, litchi PCs showed a higher absorption rate (100%) than coffee PCs (60%). These results allowed us to determine that encapsulation preserves the properties of the PCs and provides better conservation percentages than other studies, which could be a valuable addition to the functional ingredients market, offering greater value to these by-products.

## 1. Introduction

The agricultural and food sectors generate substantial amounts of waste that can be utilized to obtain value-added products, representing pivotal strategies for environmentally friendly production [1]. Nevertheless, for an extended period, agro-industrial waste has been predominantly employed in industries such as energy, agrochemistry, and livestock production, thereby constraining its utilization in applications such as biomass energy generation, biofertilizer production, animal feed production, and the manufacturing of materials and products including ethanol, essential oils, and additives [1,2,3]. However, the diverse characteristics and chemical compounds present in agro-industrial waste have rendered it a significant source for the procurement of bioactive compounds, which are of considerable importance [2]. In agro-industrial waste such as litchi peel and coffee pulp, a wide variety of compounds, alkaloids, diterpenes, flavonoids and nonflavonoids, and polyphenols, among many others, have been identified [4,5,6]. Among the flavonoid polyphenols, flavan-3-type compounds have been identified as procyanidins. These compounds are of great interest because of their many proven bioactive properties, which provide them with potential clinical or food applications, mainly because of their high antioxidant potential [7,8].

However, the effective application of these compounds in foods can face challenges related to their stability and bioavailability, and their efficacy can be compromised by various factors during digestion. Owing to the action of digestive enzymes, changes in pH and interactions with other food components may significantly influence the release and bioavailability of these compounds [8,9,10]. Hence, the need arises for the application of techniques such as microencapsulation, which has been consolidated as an effective strategy to provide greater availability [11,12] and stability to environmental conditions such as light, oxygen, or temperature [13]. The use of microencapsulation can provide greater stability even when compounds are incorporated as functional ingredients or added to food matrices. A variety of methodologies that provide significant advantages in the encapsulation of bioactive compounds exist. These include coacervation, emulsification, fluidized bed coating, and spray drying. The latter method is notable for its economic efficiency, flexibility, and high performance. Additionally, it allows for the utilization of diverse wall materials, which is a crucial factor in the effectiveness of the method [14].

The selection of suitable wall materials, such as maltodextrin (MD) and animal proteins such as whey protein (WP), may enhance the stability of the encapsulated compounds. These wall materials possess high retention capacity and emulsifying properties and provide excellent solubility, which contributes to the effectiveness of the encapsulation process. Additionally, the use of these wall materials can prevent interactions between different compounds, allowing for the adequate retention of the bioactive properties of the encapsulated compounds and facilitating their delivery to the desired target tissues [15,16].

In the case of procyanidins from coffee pulp and litchi peel, microencapsulation could preserve their stability not only during food processing and storage, but also in the gastrointestinal tract. Due to this and the application of these types of microencapsulated compounds in the food industry, it is necessary to study all the processes by which microencapsulation modifies the transformation and behavior of these compounds during the digestion process, which is required to achieve and modulate the release of procyanidins from the encapsulation matrix for absorption in the small intestine [13,15]. In this regard, in vitro digestion studies have emerged as a valuable tool for simulating gastrointestinal processes and evaluating the release of encapsulated compounds. The effects of in vitro digestion on the stability of procyanidin and phenolic compounds in general, derived from a variety of sources, have been the subject of numerous studies. Studies on the digestion of these compounds in their free form, as conducted by Ketnawa et al. [9] and Gómez-García et al. [17], have demonstrated that this process can result in the loss of various bioactive properties, including antioxidant capacity.

In this regard, the impact of diverse encapsulation techniques and wall materials on the stability of the compounds has been investigated, thereby enhancing their preservation. This is evidenced by studies such as [18,19]. Nevertheless, there is a paucity of research examining the bioaccessibility and functional stability of these compounds throughout the gastrointestinal digestion model especially focused on CPs. This limits their applicability in the development of functional foods and the comprehensive understanding of the bioavailability and bioaccessibility of bioactive compounds, which are pivotal factors in determining their capacity to exert physiological effects in organisms. This approach can provide detailed information on the resistance to digestion and the release of compounds in different segments of the gastrointestinal tract. In addition, there are no studies on the digestion of procyanidin specifically; the information that is available focuses mainly on the digestion of polyphenolic compounds in general.

Therefore, the objective of this study was to evaluate the in vitro digestion of procyanidins derived from coffee pulp and litchi peel, both of which had undergone microencapsulation. This study aims to demonstrate that microencapsulation can enhance the stability and bioactivity of procyanidins by providing a detailed understanding of the impact of different digestive phases. The study focuses on changes in concentration, antioxidant capacity, and absorption under physiological conditions. This study establishes a foundation for optimizing the utilization of these agro-industrial by-products and enhancing the functionality of bioactive compounds. Additionally, tannins and procyanidins from litchi peel and coffee pulp were characterized using UPLC-QqQ.

## 2. Materials and Methods

### 2.1. Extraction and Partial Purification of Procyanidins

The litchi fruits were collected in the Tres Marías orchard (21.476124 north, −98.975623 west), in the municipality of Huichihuayan, SLP, Mexico. The coffee pulp was obtained from local cultivars in the municipality of Xilitla (21.364526 north, −98.963230 west), SLP, Mexico. The litchi fruits and peels were separated manually, as well as the coffee pulp and beans. The material was dried in an oven at 50 °C for 48 h and then crushed in a hand mill. The extraction was carried out by placing the plant material in a solution of 70% acetone for the coffee pulp and 96% ethanol for the litchi peel, at a ratio of 1:10. The extraction was assisted with ultrasonication in a bath (Brason model 3800, Brookfield, CT, USA) for 20 min. After the extraction process was complete, the solutions were filtered and stored until use at 4 °C in completely closed containers isolated from light.

The partial purification of procyanidins was carried out on a preparative chromatograph (Büchi, Pure C-850 FlashPrep, Flawil, Switzerland). It was used in a column packed with 40 g of Sephadex LH-20. Distilled water, ethanol, and acetone were used as mobile phases. The elution method was carried out as follows: 0–10 min, 100% deionized water; 10–20 min, 100% ethanol; 20–30 min, 90% acetone and 10% water; and 30–40 min, 100% deionized water. The flow rate was 30 mL/min and the detector was set at 254 nm.

### 2.2. Microencapsulation via Spray Drying

The spray drying microencapsulation process was performed according to the technique described by Vázquez-Núñez et al. [20]. According to the method, the best encapsulating material for each sample was used to protect the procyanidins and evaluate their correct release, maltodextrin (JR Foods^®^, Monterrey, NL, Mexico, 98% purity) for litchi procyanidins and whey protein (Isopure^®^, Louisville, KY, USA, isolate 80% protein) for coffee procyanidins. In the same way, the encapsulating agent solutions were prepared according to the best percentage of material (1% *w*/*v*), and these solutions were allowed to hydrate for 24 h at 4 °C. After this time, the acetone fractions resulting from the partial purification were subjected to rotary evaporation to remove the solvent, and the volume equivalent to 1 mg of procyanidins per 100 mL of the encapsulating agent solution was added and left to stand for 1 h. The microcapsules were subsequently designed using the spray drying method (Büchi Mini spray dryer B-290, Flawil, Switzerland), using the optimal encapsulation conditions shown in Table 1.

### 2.3. Quantification of Total Procyanidins

The butanolysis method was used as described below, with modifications [21]. The reaction was carried out in tubes with 1.5 mL of a concentrated butanol (nBuOH)-HCl solution (95:5 *v*/*v*), 50 µL of a 2% ferric agent solution (prepared with NH_4_Fe (SO_4_)_2_•12H_2_O in HCI 2 M), and 250 µL of each extract and the obtained fractions containing the procyanidins. The tubes were mixed and placed in a water bath at 95 ± 0.2 °C for 40 min. After the heating time, the samples were cooled for 30 min and read with a spectrophotometer (Genesys 10S UV-Vis Thermo Scientific, Waltham, MA, USA) at 550 nm. Procyanidin B2 standard was used for quantifying the PCs content (Figure S1).

### 2.4. Quantification of Total Polyphenols and Total Flavonoids

The total polyphenol technique was used as described by Nossa González et al. [22], with some modifications. From the extract or previously released encapsulates, 125 µL of the sample was taken and placed in test tubes, and 50 µL of distilled water and 125 µL of Folin and Ciocalteu’s reagent were added. They were covered to avoid contact with light and allowed to incubate for 6 min at room temperature. Subsequently, 1250 µL of 7% Na_2_CO_3_ and 1000 µL of distilled water were added. The mixture was covered again and incubated for 90 min, after which the absorbance at 760 nm was read, and water was used as a blank. Gallic acid was used as standard (Figure S2).

Flavonoids were measured as follows: extracts of each plant material (1 mL) were diluted with 4 mL of water in a 10 mL volumetric flask. Initially, 5 % NaNO_2_ solution (0.3 mL) was added to each volumetric flask. After 5 min, 10 % (*w*/*w*) AlCl_3_ was added and after 6 min, NaOH 1.0 MH was added to each flask. NaOH 1.0 M (2 mL) was added after 6 min. The absorbance of the reaction mixture was read at 430 nm. Catechin was used as a standard and the total flavonoid content was expressed as mEq cat/mL of sample (Figure S3).

### 2.5. Antioxidant Activities

#### 2.5.1. ABTS Assay

The ABTS radical inhibition assay was performed according to the methodology proposed previously by Castro-López et al. [23], with minor modifications. The ABTS radical cation was generated from an aqueous ABTS solution (7 mM) with potassium persulfate (2.45 mM) in the dark and at room temperature for 12 h before use. Diluted ABTS solutions were prepared in ethanol to a value of 0.700 ± 0.002 absorbance units. Varying concentrations plant extracts (50 µL) were allowed to react with 950 µL of the ABTS solution, and after 1 min of reaction, the absorbance was measured at a wavelength of 734 nm. The ability to inhibit the radical (expressed as the percentage inhibition of the ABTS radical) was compared with that of Trolox as a standard (Figure S4), and calculated as Equation (1):

Inhibition (%) = (A − B)/A × 100(1)

where A is the absorbance of the control reaction (containing all the reagents except the test compound) and B is the absorbance of the sample.

#### 2.5.2. DPPH Assay

To evaluate the free radical scavenging capacity of the extracts and encapsulates, the degree of discoloration caused by their components to a methanolic solution of DPPH was determined via the method of Castro-López et al. [23], with some modifications. First, the calibration curve was generated with solutions of gallic acid at concentrations ranging from 7.8 to 2000 µg/mL. Then, 100 µL was taken and placed in test tubes with 2.9 mL of a 200 µM DPPH solution prepared in methanol. The solution was allowed to stand for 30 min in the dark. The absorbance of the mixture was measured spectrophotometrically at 517 nm. For the samples, 25 µL of the samples were taken in triplicate and placed in test tubes with 725 µL of the DPPH solution, and the same procedure was followed. The results of the antioxidant activity were expressed as µg EGA/mL, since the absorbances were compared against a gallic acid standard curve.

#### 2.5.3. Lipid Oxidation Inhibition (LOI) Assay

To quantify the ability of the extracts and encapsulates to inhibit the generation of hydroxylated peroxides in the early stages of linoleic acid oxidation, the method described by Castro-López et al. [23] was performed, with slight modifications. The reaction was performed by placing 50 µL of the sample in a test tube with 100 µL of a linoleic acid solution (0.6 g of linoleic acid and 1.5 g of Tween 20 in 8 mL of ethanol), and subsequently adding 1500 µL of 0.02 M acetate buffer solution, pH 4. The mixture was homogenized and incubated at 37 °C for 1 min. Then, 750 µL of 2.5 M FeCl_2_ (0.01 g of FeCl_2_ and 0.017 g of EDTA in 100 mL of distilled water) was added, and 250 µL of each reaction was taken to measure the absorbance at time zero. The remaining solution was incubated at 37 °C for 24 h. After the incubation time, 250 µL aliquots were taken and transferred to test tubes with 1 mL of a 0.1 M NaOH solution (prepared in 10% ethanol) to stop the oxidation process and 2.5 mL of 10% ethanol to dilute the sample. The absorbance of the mixture was measured spectrophotometrically at 232 nm, 10% ethanol was used as a blank, and a mixture of reagents without a sample was used as a control. The inhibition of linoleic acid oxidation was calculated using Equation (2):

Lipid oxidation inhibition (LOI)= (A − B)/A × 100(2)

where A is the difference between the absorbance of the control after 24 h and 0 h of incubation, and B is the difference between the absorbances of each extract sample after 24 h and 0 h of incubation.

#### 2.5.4. Oxygen Radical Absorbance Capacity (ORAC)

The assay was performed following the methodology of Ou et al. [24]. In a dark 96-well microplate, 20 μL of the sample and 200 μL of fluorescein at 1.09 µM were added and allowed to incubate for 15 min at 37 °C. After incubation, a first reading was taken, then 75 μL of 2,2-azobis(2-methylpropionamidine) dihydrochloride (AAPH) was added to each well, and the plate was read at 210 s intervals for 120 min at 485 nm for excitation and 580 nm for emission wavelengths. The area under the curve was obtained using SigmaPlot software version 12.0.

#### 2.5.5. Ferric Reducing Antioxidant Power Test (FRAP)

The FRAP assay was carried out according to the methodology described by Ghasemzadeh et al. [25]. The radical mixture was prepared by mixing 400 mM acetate buffer, 30 mM 2,4,6-tripyridyl-s- triazine (TPTZ), and 60 mM ferric chloride hexahydrate at a ratio of 1:1:10 (*v*/*v*/*v*). In a clear, 96-well microplate, 10 μL of sample mixture and 190 μL of reagent mixture were mixed, shaken, and allowed to stand for 20 min in the dark. The absorbance was read at 593 nm. Trolox was used as standard solution (Figure S5).

### 2.6. Phytochemical Characterization and Quantification Using UPLC-PDA-ESI-QqQ

The extracts and purified fractions were analyzed for the identification and quantification of flavonoid compounds and phenolic acids according to the UPLC-PDA-ESI-QqQ method of Díaz-Rivas et al. [26]. One milliliter of each sample was dried in a rotary evaporator, subsequently resuspended in 2 mL of methanol, and filtered through 0.45 µm PTFE filters prior to analysis. The UPLC system consisted of an Acquity class-H quaternary pump (QSM-”quaternary solvent module”) and sample handler (SM-FTN) (Waters Corp., Milford, MA, USA) coupled to a Xevo TQ-S triple quadrupole tandem mass spectrometer (Waters Corp.). The data were recorded in multiple reaction monitoring (MRM) mode. Data acquisition and processing were performed with MassLinx version 4.1 software (Waters Corp.). Chromatographic separations were performed on a C18 Acquity UPLC BEH (100 mm × 2.1 mm × 1.7 µm) column (Waters Corp.) operating at 35 °C, with 7.5 mM water/formic acid (A) and acetonitrile (B) as mobile phases at 0.35 µL/min and a sample volume of 2 µL. A solvent gradient was applied, starting with 3% B, maintaining the flow for 1.23 min, followed by 9% B until 3.82 min, 16% B until 11.40 min, 50% B until 13.24 min, 3.0% B until 15 min, and then a return to the initial conditions (3% B). Negative ionization was used for the MS assay. The ESI conditions were as follows: capillary voltage, 2.5 kV; desolvation temperature, 300 °C; temperature source, 150 °C; desolvation and cone gas, 500 L/h and 151 L/h, respectively; and collision gas, 0.13 mL/min. MRM transitions were determined from the MS/MS spectra of the existing phenolic acid standards, and a mixture of different phenolic compounds was used as a monitor for retention time and m/z values. The identification of the peaks was based on the comparison of their retention times and MRM transitions with those of the pure standards. The quantitative determination of phenolic compounds was carried out via calibration curves of the available standards.

### 2.7. Characterization of Procyanidins

#### 2.7.1. Phloroglucinolysis Reaction

Fifty milligrams of lyophilized extracts of litchi peel and coffee pulp and 800 µL of a phloroglucinol solution (50 g/L phloroglucinol, 10 g/L ascorbic acid, 0.1 N HCL) were used, vortexed, and incubated for 20 min in a bath at 50 °C. Then, the reaction was stopped by placing the vials on ice, and the mixture was diluted with 1 mL of 40 mM sodium acetate. The mixture was subsequently centrifuged at 4500 rpm for 10 min, after which the supernatant was recovered and filtered through a 0.45 µm filter. Finally, the aliquot was dispersed in an amber vial and injected into the UPLC-PDA-ESI-QqQ.

#### 2.7.2. UPLC-PDA-ESI-QqQ Analysis

The chromatographic conditions used were as follows: an Acquity UPLCr BEH C18 1.7 µm × 59 mm × 2.1 mm column, 1 µL injection volume, 40 °C column temperature, 10 °C sample temperature, 4.5 min run time, Phase A: water 2.5% acetic acid and Phase B: acetonitrile. A gradient was applied starting with 3% B, maintaining the flow rate at 0.61 mL, followed by 9% B until 0.43 min, 16% B until 1.02 min, 50% B until 3.06 min, 3% B until 3.54 min, and the initial conditions (3% B) until 4.00 min. The chromatograms were recorded at 280 nm, and a sweep was programmed from 240 to 600 nm to determine the spectral pattern of the compounds. A fitted line was generated with catechin and epicatechin at concentrations ranging from 0 to 20 ng. The QqQ conditions were as follows: ESI- polarity, capillarity 2.50 kV, cone 30 V, 60 V output source, 60 °C output temperature, source temperature 150 °C, cone gas flow rate 150 L/h, collision gas flow rate 0.13 mL/min, nebulizer gas flow 7.00 bar, resolution LM 1: 2.8, resolution HM 1: 14.9, ion energy 1:0.6, resolution LM 2: 2.8, resolution HM 2: 14.9, ion energy 2: 1.0, MS/MS mode collision energy: 20, and mass scan at 100–1100 Da. The mean degree of polymerization (mDP) was calculated as the sum of flavan-3-ol monomers and phloroglucinol adducts divided by the sum of the monomers.

### 2.8. In Vitro Digestion

To evaluate the effects of digestion on microencapsulated procyanidins and the stability of bioactive compounds, an in vitro digestion simulation was conducted according to the method described by Sanchez-Gutierrez et al. [27], with modifications. Samples were prepared in triplicate in two independent experiments, each consisting of 500 mg of microencapsulates mixed with 10 mL of water. The digestion process was simulated by replicating physiological conditions, including pH and temperature changes, peristaltic movements, enzyme activity, and the addition of bile salts from a pharmaceutical source. Intestinal absorption was simulated using a dialysis process. All enzyme solutions were freshly prepared prior to use.

The simulation was performed using a shaker set at 37 °C with constant mechanical agitation to mimic body temperature and peristaltic movements. At the end of each digestion phase, 1 mL aliquots of the digestion mixture were collected and frozen for later analysis of bioactive compound content and antioxidant capacity. These were assessed before and after each digestion stage using the HCL-butanol assay and ABTS, DPPH, and lipoperoxidation techniques, as previously described.

#### 2.8.1. Simulated Oral Digestion

The initial pH of each sample was adjusted to between 5.6 and 6.9 using 1 M HCl or 0.1 M NaOH. Buccal digestion was performed with 0.3 mL of α-amylase solution (100 U/mL and CaCl_2_, 1 mM), and the mixture was incubated for 2 min at 200 rpm.

#### 2.8.2. Simulated Digestion in the Stomach

For gastric digestion, the pH of the samples was adjusted to 2.0 using 1 M HCl. A pepsin solution (25 mg/mL in HCl 0.1 N) was added at a rate of 0.05 mL/mL to simulate gastric juice. The mixture was incubated for 1 h under agitation at 130 rpm.

#### 2.8.3. Simulated Intestinal Digestion

Digestion in the small intestine was performed by adjusting the pH to 6.0 with 1 M NaHCO_3_. Intestinal juice was simulated by dissolving 1.338 g of Espaven^®^ in 100 mL of 0.1 M NaHCO_3_. The mixture was added at a concentration of 0.25 mL/mL sample. The solution was then incubated for 2 h at 45 rpm.

### 2.9. In Vitro Intestinal Absorption

For the simulation of procyanidin absorption, a membrane (6–8 kDa) in the shape of an elongated cylinder 3 cm wide was used, from which segments were cut, leaving 10–20% extra length for air space to ensure buoyancy. The membrane was placed to hydrate in distilled water at room temperature for 30 min to remove the preservative and then rinsed with distilled water. To set up the dialysis system, a beaker was filled with 500 mL of the dialysate solution (50 mM phosphate buffer, pH 7–7.5); the appropriate volume should be in a 1:100 ratio of sample volume to dialysate solution. Subsequently, the previously rinsed dialysis membrane was taken, and one end was sealed with plastic clamps 3–5 cm from the bottom. Subsequently, through the open end, 5 mL of the sample resulting from the digestion (after the 3 stages) was loaded into the membrane simulating the small intestine and the top of the membrane was secured at least 5 cm from the end, leaving enough space for flotation. The membrane was placed in the vessel with the phosphate solution and kept at 150 rpm agitation at 37 °C. Aliquots of 500 µL of the “OUT” fraction (outside the membrane) were taken at 1, 24, and 48 h of dialysis to quantify the procyanidin content with the above-mentioned technique; this fraction represents the absorbable fraction. Samples were kept in the absence of light and frozen until analysis.

## 3. Results

### 3.1. Bioactive Compounds and Antioxidant Capacity

The flavonoid, polyphenol, and procyanidin contents of the crude extracts and the fractions obtained from the purification process are shown in Table 2. After the purification process and quantification of the three bioactive compounds for both samples, the highest recovery rate was obtained in the acetone fraction, since the concentrations recovered in the ethanol fraction were significantly lower, so this fraction was discarded for further analysis. Based on the procyanidin content per mL of extract, the agro-industrial residues were found to have a concentration of 1537.09 mg and 1260.81 mg of PCs per 100 g of dry plant material for litchi peel and coffee pulp, respectively, representing 1.5 and 1.2% of the dry weight, respectively. Finally, the antioxidant activity evaluated in the crude extracts and in the acetone fraction is shown in Table 3, where it is observed that in all the results of the litchi samples and in most coffee samples, the acetone fraction has a significantly lower antioxidant activity than the crude extract.

### 3.2. Characterization of Phytochemicals Using UPLC-PDA-ESI-QqQ

The results of the analysis revealed the presence of a total of 11 flavonoids and 9 phenolic acids, identified via UPLC-PAD/ESI-QqQ MS/MS analysis according to their main ionic transitions and retention times, in addition to their molecular weights and λ max values. Some differences were observed in the profiles of the different samples (Table 4), mainly between the extracts and the already purified fractions, since 11 flavonoids and 9 phenolic acids were identified in the coffee extract, of which only 9 flavonoids and 5 phenolic acids were retained in the purified fraction. In the case of litchi extract, out of 11 flavonoids and 9 phenolic acids, only 7 flavonoids and 6 phenolic acids were retained in the purified fraction.

### 3.3. UPLC-ESI-QqQ Characterization of Procyanidins

The profiles of the flavan-3-ols are shown in Table 5. The compounds were identified on the basis of the retention times (RT), transition, and maximum absorbance of the standards. This analysis allowed for broader characterization of the flavan-3-ol compounds present in the samples, such as the presence of procyanidin B2 in both extracts and B1 only in the coffee sample. However, it is possible to observe that the highest concentration of flavan-3-ol type bioactive compounds was much more abundant in the litchi peel samples, which agrees with previous analyses, while epicatechin (EC) is the main compound in both extracts (RT 3.51, transition 289 > 203 > 123 and λ Max 278.86 nm) and the other compounds are present in much lower concentrations. The content of flavan-3-ol monomers and phloroglucinol adducts showed mDP values of 1.5 and 1.7 for coffee and litchi, respectively.

### 3.4. Changes Produced in Microencapsulates During In Vitro Digestion

**Figure 1:**
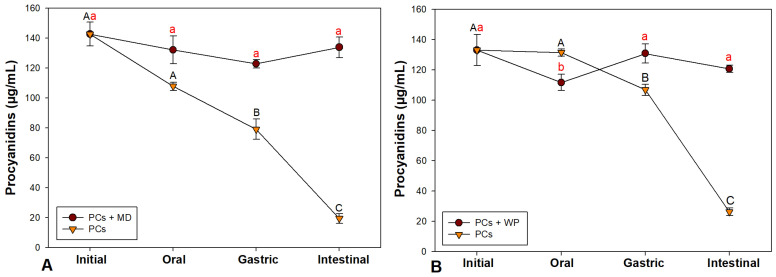
Changes in the concentration of procyanidins during in vitro digestion: (**A**) procyanidins from litchi; (**B**) procyanidins from coffee; PCs + MD = procyanidins encapsulated with maltodextrin; PCs + WP = procyanidins encapsulated with whey protein; and PCs = free procyanidins. Different letters indicate significant differences using Tukey’s test (*p* ≤ 0.05).

Figure 1 shows the changes produced in the concentration of encapsulated procyanidins (PCs + MD and PCs + WP) and free procyanidins (PCs) during the different phases of digestion. Taking as a reference the concentration of the encapsulated litchi and coffee procyanidins before the in vitro digestion process, the results showed a final yield (after the intestinal phase) of 94.81 ± 4.83% and 90.74 ± 1.77% of litchi and coffee procyanidins, respectively, in contrast to the free procyanidins, which suffered a significant progressive decrease during the digestion process, with a final yield of only 13.71 ± 2.25% and 19.80 ± 1.80%, respectively.

### 3.5. Absorption of Procyanidins

**Figure 2:**
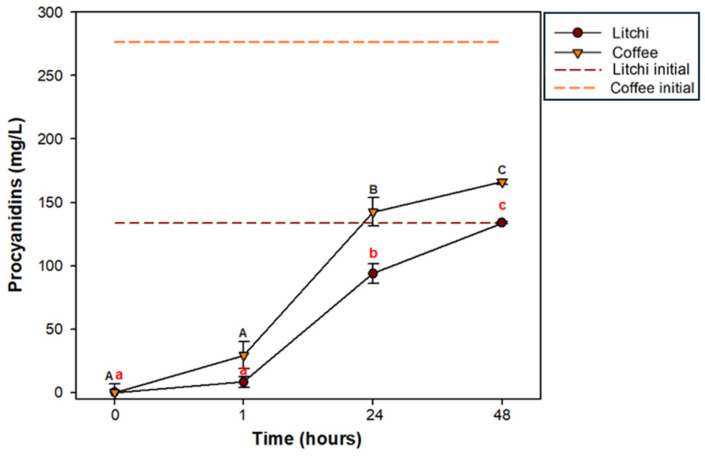
Absorption of procyanidins from litchi and coffee. The initial concentration of procyanidins placed inside the membrane is represented in both cases with a dotted line. Different letters indicate significant differences using Tukey’s test (*p* ≤ 0.05).

As shown in Figure 2, the concentration of procyanidins outside the membrane recalculated as a function of the volume used for the absorption simulation showed a continuous increase with time as they accumulated in the external medium, reaching a maximum at 48 h, simulating the passage of procyanidins through the wall of the small intestine. At this time, the total procyanidins digested from litchi reached 100% absorption. In contrast, procyanidins digested from coffee showed limited absorption, and the total absorbed procyanidins represented a rate of 60%. At the same time, although the concentration of procyanidins from coffee is considerably higher compared to litchi, the percentage is derived from the initial total concentration placed inside the membrane, which was not achieved in the case of coffee.

### 3.6. Changes in Antioxidant Activity During In Vitro Digestion

**Figure 3:**
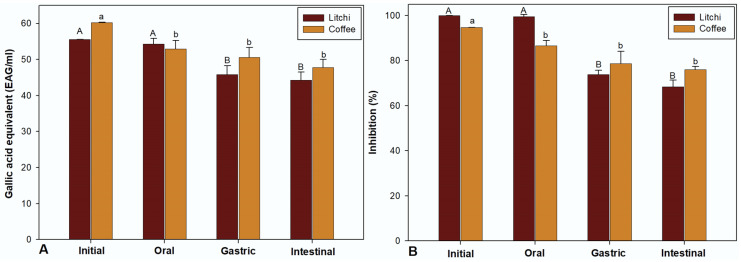
Changes in the antioxidant capacity of litchi and coffee procyanidins during in vitro digestion: (**A**) DPPH; (**B**) lipid oxidation inhibition. Along the rows, different letters indicate significant differences using Tukey’s test (*p* ≤ 0.05).

The antioxidant capacity of coffee PCs also remained stable during all phases of digestion, as shown in Figure 3, unlike that of litchi PCs, which showed a statistically significant decrease after the gastric phase; however, after this phase, the activity was maintained, resulting in a final conservation percentage (after the intestinal phase) of the antioxidant capacity of coffee PCs of 79.29% and 75.96% and of litchi PCs of 79.69% and 68.33%.

## 4. Discussion

### 4.1. Extraction, Purification, and Characterization of Procyanidins

It has always been of utmost importance to adequately preserve the compounds of interest during extraction and purification processes. In the purification of bioactive compounds, although both solvents allow for the recovery of this type of compounds, acetone showed a greater recovery capacity, due to its high affinity attributed mainly to its polarity, an effect that has been shown in studies such as that of Rodrigues et al. [28] in the recovery of phenolic compounds with different solvents including methanol and butanol. Furthermore, despite a decrease in the concentration of procyanidins, flavonoids, and polyphenols in the acetone fraction with the purification process, the quantified concentrations remain representative. Hence, the concentrations and percentages by weight of coffee pulp are in the ranges reported in the literature. These values are consistent with those reported in the literature, including average percentages of 0.8 and 2.88% in the pulp of *Coffea arabica* and *Coffea canephora*, respectively [29], and the range of condensed tannin concentrations in coffee pulp, which has been documented to vary between approximately 1000 and 2000 mg/100 g [30]. In contrast, these results are significantly higher than those reported by other researchers, who have documented maximum values between 610 and 679 mg/100 g [30,31].

In the case of litchi, the results were found to fall within the ranges reported by authors such as Zu et al. [5], who mention percentages of different types of procyanidins of between 1% and 1.6%. However, in the percentages in dry weight, other studies have reported percentages higher than those obtained experimentally, reaching up to 6.9% [32] and 285.3 mg/g [33]. It is acknowledged that discrepancies may arise between the concentrations of bioactive compounds identified in different investigations of the same plant material. These inconsistencies have been attributed to a range of environmental factors, including temperature, geographical location, level of irradiation, soil type, climate, and plant stress [34]. It is important to note that even these materials, which are agro-industrial residues, present higher concentrations than some foods that are common sources of procyanidins, such as red wine (313 mg/L), blueberries (331.9 mg/100 g), and apples (65 mg/100 g) [35].

Conversely, the statistically significant reduction in the antioxidant capacity of the purified fractions can be primarily attributed to the decline in the initial concentration of the compounds present in the extracts throughout the purification process. Consequently, the antioxidant capacity is contingent upon the totality of the compounds present. Nevertheless, the findings pertaining to the purified fractions indicate that their antioxidant capacity remains comparable to that documented in the literature by various researchers for extracts derived from these same agro-industrial by-products [35,36].

The UPLC-PAD/ESI-QqQ MS/MS analysis enabled the identification of alterations in the composition of compounds across the diverse samples. These changes were predominantly attributed to the purification process, which resulted in the elimination of and reduction in the majority of compounds in the extracts. The coffee pulp was found to contain primarily hydroxycinnamic acids, followed by hydroxybenzoic acids. Chlorogenic acid was identified as the most abundant hydroxycinnamic acid, while caffeic acid exhibited a markedly lower concentration. This discrepancy is likely attributed to the distribution of these compounds within the pulp. These findings align with those previously reported by Aristizábal et al. [37], who noted that caffeic acid is predominantly present in the coffee bean, rather than in the pulp. Moreover, these findings align with those of previous studies that have identified hydroxycinnamic acids and hydroxybenzoic acids as among the most prevalent classes of phenolic compounds in coffee pulp [38]. However, data on the composition of coffee pulp may vary due to the characteristics of coffee fruits, such as the type of crop, place of production, pulping, and degree of maturity, among many other factors, including the analytical methods used. In litchi peel, the predominant phenolic compounds were identified as hydroxybenzoic acids, with shikimic acid being particularly abundant. Flavonoids, including flavan-3-ols and flavonols, were also present in notable quantities. These findings align with the prevailing literature on litchi peel composition, as numerous studies have demonstrated that flavonols and flavan-3-ols, such as procyanidins and catechins, are the primary phenolic compounds in litchi peel [6,33].

In this case, the targeted study did not allow for a comprehensive exploration of flavan-3-ols. Therefore, a characterization analysis and the quantification of PCs, as well as a determination of the mDP by phloroglucinolysis, were developed. The mDP values indicate that both extracts possess mainly small-structure compounds; however, this result was considerably lower than that reported in studies where the mDP values reached up to 5 [32]. Furthermore, dimer-type procyanidins were identified in both samples, whereas other studies have identified procyanidins with degrees of polymerization of up to pentamers [10,37].

### 4.2. Microencapsulates Bioavailability and Absorption

The results of the in vitro digestion experiment indicate that the encapsulated PCs maintain a stable concentration, which suggests that the encapsulation process provides a protective effect for the digested compounds. This same effect has been previously reported by studies such as those of Lang et al. [18] and Toro-Uribe et al. [19], which are detailed in Table 6, where a comparison is made of the different results that can be obtained in the digestion of bioactive compounds when they are encapsulated or not.

The findings of these studies indicate that bioactive compounds exhibited enhanced preservation and stability during digestion when encapsulated. Moreover, the preservation of up to 94 and 96% of polyphenolic compounds and proanthocyanidins has been demonstrated with materials such as maltodextrin and whey protein during digestion in studies such as [11,39], respectively.

**Table 6:** Comparison of the effect of encapsulation of bioactive compounds on their stability, antioxidant activity, and absorption after an in vitro digestion process.

Compound	Concentration	Antioxidant Activity	Absorption	Study
Encapsulated litchi PCs	94%	68–77%	100%	This study
Encapsulated coffee PCs	90%	75–76%	60%	
Free anthocyanins	23–24%	Significant decrease	Significant decrease	[18]
Encapsulated anthocyanins	29–40%	Significant decrease	Improved absorption	
Free PCs	38%	43–81%		[19]
Encapsulated PCs	67%	82–91%		
Encapsulated polyphenols	58–94%			[11]
Encapsulated anthocyanins	27–51%			
Free polyphenols	45–77%	31–69%	40–80%	[9]
Free polyphenols	40%	70%	32.8%	[40]
Encapsulated proanthocyanidins	37–96%	Significant decrease		[39]

The results shown are the percentage of conservation of the concentration and antioxidant activity of the bioactive compounds compared to the value prior to in vitro digestion.

On the other hand, the final yields obtained with PCs + MD and PCs + WP were markedly higher than those reported by numerous authors who evaluated the digestion of bioactive compounds without employing any preservation process. In such instances, a notable reduction in yield was frequently observed, as illustrated in Table 6 for the studies conducted by Ketnawa et al. [9] and Mashiota et al. [40]. In the absence of encapsulating materials to safeguard the bioactive compounds, the inherent nature of the compounds and their degree of polymerization, which varies considerably, give rise to notable discrepancies between studies in terms of the percentage yields. It has been demonstrated that digestive enzymes do not exert any influence that could potentially result in the degradation of the compounds. Nevertheless, research has demonstrated that the modifications observed in these compounds during the digestive process appear to be predominantly associated with factors such as pH and the physicochemical actions of peristaltic movements within the digestive tract [19].

In the results of antioxidant capacity conservation, despite showing a decrease, the final results were significantly higher than those reported by other authors, who observed a notable decline in the antioxidant capacity of bioactive compounds in their free form during digestion. In some instances, the antioxidant capacity was reduced by over 50% following the in vitro digestion process [9,38,40]. Therefore, it can be concluded that encapsulation is an effective method for maintaining the antioxidant capacity of bioactive compounds. It is of paramount importance to ensure that the bioaccessibility of procyanidins to circulation is maintained, as this is a crucial factor in enabling the bioactive compounds to reach the target organs or tissues, where they can fulfill their functions. The stability of the compounds following digestion, their appropriate release from the encapsulation matrix, and efficient transepithelial passage are factors that influence this process [9]. Consequently, any unabsorbed procyanidins reach the colon, where they undergo microbial transformation and degradation processes. Although coffee procyanidins are considerably less accessible than litchi procyanidins, this value remains within the ranges reported by other authors, such as Ketnawa et al. [9]. Furthermore, the impact of compound enhancement through encapsulation is evident, as shown by the elevated absorption rates observed in this study and corroborated by Lang et al. [18], who demonstrated that encapsulated anthocyanins exhibit superior absorption compared to their free form, in comparison with studies such as [40], which indicate that only approximately one-third of free polyphenols are absorbable in the intestine.

On the other hand, the discrepancies in the absorption percentages of litchi and coffee procyanidins can be attributed to various factors, primarily structural in nature. The absorption of procyanidins is constrained by their degree of polymerization, a phenomenon that has been corroborated by previous research studies [19]. The degrees of polymerization of the procyanidins of litchi and coffee, mainly because they are different plant materials, may differ structurally in relation to this, as determined above. Although both extracts presented a relatively low PDM, the PDM of litchi is lower (1.2) than that of coffee (1.7). In this instance, procyanidins with elevated degrees of polymerization would be more challenging to hydrolyze into smaller, more accessible molecules. Consequently, the size threshold of the dialysis membrane may impede the passage of larger molecules [40]. Furthermore, the low uptake of procyanidins may be attributed to the type of encapsulation matrix. The action of enzymes during digestion affects the structure and stability of the matrices; however, the whey protein used as a wall material to encapsulate the procyanidins in coffee pulp is much more complex and larger in size, which may have an impact on the processes. This may result in the procyanidins binding to fragments of this matrix in the dialysis phase, which could hinder their absorption.

### 4.3. Implications and Application Potential

The detailed characterization and the high levels of stability and absorption observed position this approach as a competitive and value-added alternative, with the potential to transform agricultural by-products into high-demand ingredients in functional and therapeutic markets. The preservation of the bioactivity and stability of CPs during simulated gastrointestinal digestion allows for their inclusion in functional products with health benefits, such as fortified foods and dietary supplements. This improves their antioxidant profile and their ability to prevent diseases associated with oxidative stress. This advance offers an opportunity to develop functional ingredients that take advantage of agricultural by-products, thereby contributing to a circular and sustainable economy. Furthermore, their high retention and absorption would allow for the optimization of the bioactive benefits to consumers. In the pharmaceutical industry, these encapsulated compounds could serve as the basis for new nutraceuticals aimed at the prevention and management of different diseases.

### 4.4. Limitations of the Study

While this study offers valuable insights into the encapsulation and bioactivity of PCs derived from litchi peel and coffee pulp, it is not without significant limitations. The study focused on a limited number of functional activities, which represents a limitation of the research. It is recommended that future research should evaluate a wider range of bioactivities and their relationship to stability during digestion, in order to provide a more complete picture of the functional potential of encapsulates. Furthermore, the in vitro digestion model, while useful for simulating digestion conditions, does not fully reflect the complexity of the human digestive system. This includes interactions with the gut microbiota, which may significantly influence bioaccessibility and bioactivity. Furthermore, demonstrating the efficacy of microcapsules in preserving the characteristics of CPs in diverse food matrices through the assessment of interactions with other components could significantly contribute to their validation. Future studies could address these limitations to validate the results and optimize commercial applications of encapsulated CPs as functional ingredients.

## 5. Conclusions

The UPLC-PDA-ESI-QqQ method allowed for the identification of 27 different phenolic compounds, including the presence of 2 types of dimeric procyanidins in the extracts of agro-industrial residues of litchi and coffee, of which shikimic acid and chlorogenic acid, respectively, and epicatechin in both extracts, were the most abundant. In addition to these compounds, litchi peel and coffee pulp are important sources of procyanidins that can be recovered with procyanidin dry weight percentages of 1.5 and 1.2%, respectively. Furthermore, these types of materials subjected to the spray drying microencapsulation process can adequately retain procyanidin content and antioxidant capacity after in vitro digestion, with yields of up to 94 and 90% for litchi and coffee procyanidins, respectively. Furthermore, after changes in the procyanidins during in vitro digestion, litchi procyanidins have a higher absorption rate (100%) than coffee procyanidins (60%), mainly due to the size of the molecules determined by their mDP, 1.2 and 1.7, and the complexity in the MD and PS encapsulation matrix, respectively. However, these absorption percentages are relatively high, so these encapsulated products could constitute a strategy for their possible application as functional ingredients and an alternative to take advantage of these residues, providing them with added value.

## Figures and Tables

**Table 1:** Optimal encapsulation conditions of litchi and coffee agro-industrial waste extracts described by Vázquez-Núñez et al. [20].

Parameter	Litchi	Coffee
Feed Flow Rate mL/min	6	4.5
Inlet Air temperature °C	150	175
Inlet Air Flow m^3^/h	35	40

**Table 2:** Content of phytochemicals in extracts and flash HPLC fractions of litchi peel and coffee pulp.

Sample		Flavonoids	Polyphenols	Procyanidins
		mEq cat/mL	µg EAG/mL	µg PC/mL
Litchi peel	Crude extract	643.5 ± 86.4 ^a^	4651.21 ± 10.23 ^a^	1537.09 ± 3.57 ^a^
	Ethanol fraction	ND	229.12 ± 2.32 ^c^	203.69 ± 0.12 ^c^
	Acetone fraction	224.00 ± 2.00 ^b^	1670.90 ± 6.77 ^b^	1310.2 ± 0.97 ^b^
Coffee pulp	Crude extract	365.50 ± 20.90 ^b^	3806.11 ± 11.22 ^d^	1260.81 ± 3.67 ^b^
	Ethanol fraction	ND	115.14 ± 3.45 ^f^	78.24 ± 0.98 ^e^
	Acetone fraction	117.87 ± 12.65 ^c^	1623.25 ± 2.78 ^e^	1128.3 ± 2.12 ^d^

ND = not detected; mEq cat = concentration equivalent to mg of catechin; µg EAG = concentration equivalent to µg of gallic acid; and PC = procyanidins. Different letters in the same column indicate significant differences using Tukey’s test (*p* ≤ 0.05).

**Table 3:** Antioxidant capacity of extracts and purified fractions of litchi and coffee agro-industrial residues.

Sample		LOI	DPPH	ABTS	FRAP	ORAC
		Inhibition (%)	µg EGA/mL	mEqTrolox/mL	mEqTrolox/mL	mEqTrolox/mL
Litchi peel	Crude extract	80.50 ± 0.27 ^a^	60.90 ± 0.13 ^a^	47.62 ± 0.03 ^a^	134.54 ± 2.42 ^a^	3080.7 ± 73.49 ^a^
	Acetone fraction	67.60 ± 0.77 ^b^	59.40 ± 0.82 ^b^	21.84 ± 1.34 ^b^	54.56 ± 0.34 ^b^	1739.2 ± 110.42 ^b^
Coffee pulp	Crude extract	78.60 ± 0.55 ^a^	61.10 ± 0.14 ^ac^	45.54 ± 0.96 ^a^	142.52 ± 1.40 ^a^	3545.7 ± 96.15 ^c^
	Acetone fraction	55.3 ± 0.39 ^b^	53.10 ± 0.98 ^c^	40.79 ± 0.17 ^a^	49.93 ± 0.58 ^b^	1218.10 ± 16.30 ^d^

LOI = lipid oxidation inhibition; µg EGA = equivalent to µg of gallic acid; and mEqTrolox = milli equivalent to Trolox. Different letters in the same column indicate significant differences using Tukey’s test (*p* ≤ 0.05).

**Table 4:** Content of phytochemicals in coffee pulp and litchi peel obtained from extracts and chromatographic acetone fraction.

Compounds	Coffee Pulp		Litchi Peel	
	Extract	Acetone Fraction	Extract	Acetone Fraction
Hydroxycinnamic acids				
Quinic acid	48.70 ± 1.37 ^a^	TR	7.04 ± 0.79 ^b^	TR
Chlorogenic acid	52.95 ± 7.02 ^a^	TR	TR	TR
Caffeic acid	0.089 ± 0.00 ^a^	ND	TR	ND
Caffeoylquinic acid	48.11 ± 7.13 ^a^	0.14 ± 0.06 ^b^	0.18 ± 0.04 ^b^	0.66 ± 0.19 ^c^
Dipheoylquinic acid	17.82 ± 0.70 ^a^	ND	3.75 ± 0.03 ^b^	ND
Hydroxybenzoic acids				
Shikimic acid	0.65 ± 0.00 ^a^	0.71 ± 0.08 ^a^	54.91 ± 2.61 ^b^	4.19 ± 0.81 ^c^
Gallic acid	12.22 ± 0.58 ^a^	ND	0.11 ± 0.01 ^b^	ND
Benzoic acid	3.43 ± 0.83 ^a^	0.66 ± 0.44 ^b^	0.34 ± 0.01 ^b^	1.88 ± 0.5 ^a^
4-hydroxybenzoic acid	TR	ND	TR	TR
Flavan-3-ols				
Procyanidin B2	TR	TR	4.30 ± 0.18 ^a^	TR
Epicatechin	TR	TR	18.05 ± 1.92 ^a^	TR
Catechin	TR	TR	TR	TR
Flavonols				
Rutin	1.11 ± 0.16 ^a^	TR	14.32 ± 1.86 ^b^	0.121 ± 0.06 ^c^
Quercetin	0.02 ± 0.00 ^a^	TR	0.17 ± 0.05 ^b^	TR
Taxifolin	TR	TR	TR	TR
Quercetin-3-O-glucoside	0.209 ± 0.01 ^a^	TR	0.760 ± 0.14 ^a^	TR
Flavanones				
Naringenin	TR	ND	TR	ND
Naringin	TR	ND	TR	ND
Dihydrochalcones				
Hesperidin	0.627 ± 0.23 ^a^	TR	TR	ND
Phlorizin	TR	TR	TR	ND

Results are shown as average of three determinations ± standard deviation in µg/mL of sample. TR = trace concentration, ND = not found. Values with different letters indicate significant differences between each sample, determined using Tukey’s test (*p* < 0.05).

**Table 5:** Procyanidin content and characterization of the phloroglucinolysis reaction of coffee pulp and litchi peel extracts.

Compound	RT (min)	Transitions (m/z)	λ Max (nm)	Coffee µg/mg	Litchi µg/mg
Catechin (C)	2.8	289 > 203 > 123	278.86	0.0268 ± 0.000	0.079 ± 0.003
Epicatechin (EC)	3.51	289 > 203 > 123	278.86	1.5521 ± 0.146	4.447 ± 0.543
(−)-Epicatequina galato (ECG)	4.56	441 > 289 > 169	266.86	0.032 ± 0.001	ND
(+)-Gallocatechin (GC)	1.37	305 > 179 > 124.9	266.86	0.009 ± 0.000	0.0015 ± 0.000
(−)-Epigallocatechin (EGC)	7.32	305 > 179 > 124.9	276.86	0.026 ± 0.001	0.045 ± 0.005
Catechin 4-phloroglucinol (C-PHL)	2.07	413.3 > 261.1 > 125	277.86	ND	0.118 ± 0.011
Epicatechin 4-phloroglucinol (EC-PHL)	2.21	413.3 > 261.1 > 125	276.86	1.670 ± 0.120	5.337 ± 0.281
Procyanidin B1	2.52	577.1 > 425.1 > 289	270.86	0.029 ± 0.002	ND
Procyanidin B2	3.22	577.1 > 425.1 > 289	280	0.437 ± 0.055	2.276 ± 0.498
Unknown	4.54	577.1 > 425.1 > 290	265.86	0.089 ± 0.013	1.429 ± 0.248
(+)-Catechin gallate (CG)	5.36	441 > 289 > 169	275.86	0.039 ± 0.003	ND
Mean degree of polymerization (mDP)				1.7 ± 0.040	1.2 ± 0.030

Results are shown as average of three determinations ± standard deviation in µg/mg of sample. ND = not detected.

## Data Availability

The datasets used in the current study are available from the corresponding authors upon reasonable request.

## Supplementary Materials

The following supporting information can be downloaded at: https://www.mdpi.com/article/10.3390/polym17050687/s1. Figure S1: ABTS calibration curve; Figure S2: FRAP calibration curve; Figure S3: Procyanidins calibration curve; Figure S4: Total polyphenols calibration curve; Figure S5: Flavonoids calibration curve.
