# Supplementary material for: Biopolymer-Based Microencapsulation of Procyanidins from Litchi Peel and Coffee Pulp: Characterization, Bioactivity Preservation, and Stability During Simulated Gastrointestinal Digestion
Source: Polymers (Basel). 2025 Mar 4;17(5):687. doi: 10.3390/polym17050687 (PMC11902710; doi:10.3390/polym17050687)
Supplement: Supplementary file 1 [file polymers-17-00687-s001.zip › polymers-3399096-supplementary.pdf]

Supplementary material

Calibration curves

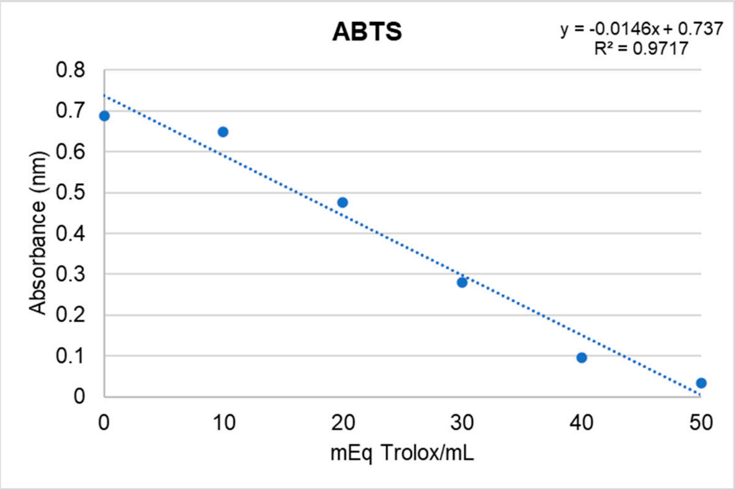

Figure S1. ABTS calibration curve.

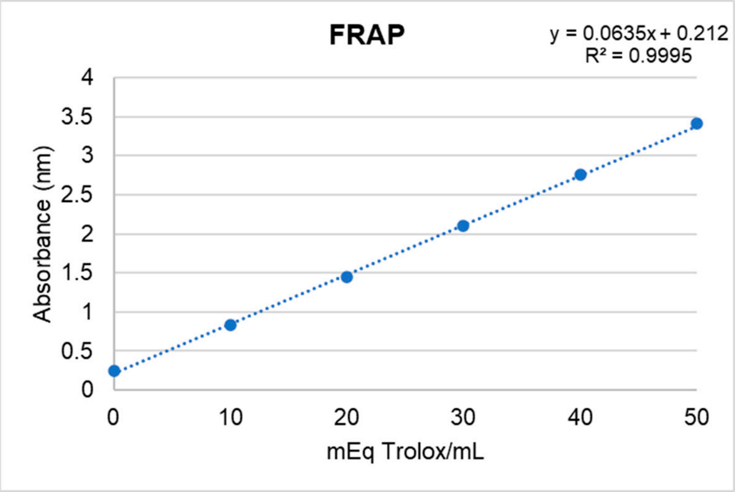

Figure S2. FRAP calibration curve.

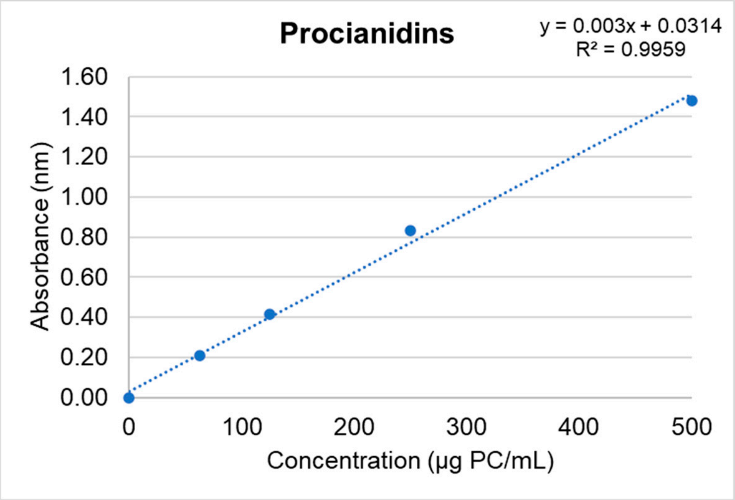

Figure S3. Procyanidins calibration curve.

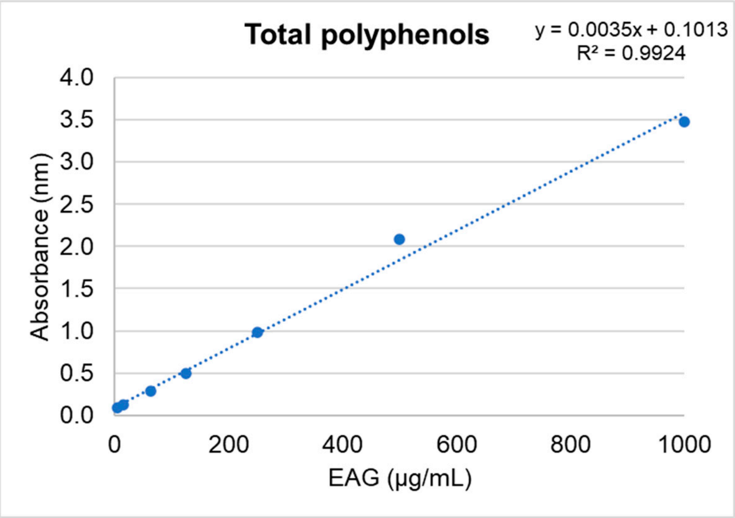

Figure S4. Total polyphenols calibration curve.

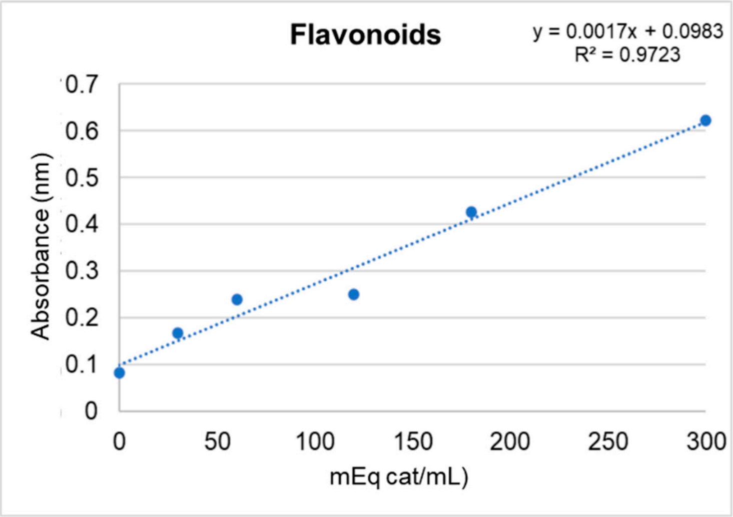

Figure S5. Flavonoids calibration curve.
